# Species Richness, rRNA Gene Abundance, and Seasonal Dynamics of Airborne Plant-Pathogenic Oomycetes

**DOI:** 10.3389/fmicb.2018.02673

**Published:** 2018-11-15

**Authors:** Naama Lang-Yona, Daniel A. Pickersgill, Isabel Maurus, David Teschner, Jörn Wehking, Eckhard Thines, Ulrich Pöschl, Viviane R. Després, Janine Fröhlich-Nowoisky

**Affiliations:** ^1^Multiphase Chemistry Department, Max Planck Institute for Chemistry, Mainz, Germany; ^2^Institute of Molecular Physiology, Johannes Gutenberg University Mainz, Mainz, Germany; ^3^Institute for Microbiology and Wine Research, Johannes Gutenberg University Mainz, Mainz, Germany

**Keywords:** airborne Oomycetes, Peronosporomycetes, plant pathogen, seasonal distribution, Sanger sequencing, qPCR analysis, meteorological parameter

## Abstract

Oomycetes, also named Peronosporomycetes, are one of the most important and widespread groups of plant pathogens, leading to significant losses in the global agricultural productivity. They have been studied extensively in ground water, soil, and host plants, but their atmospheric transport vector is not well characterized. In this study, the occurrence of airborne Oomycetes was investigated by Sanger sequencing and quantitative PCR of coarse and fine aerosol particle samples (57 filter pairs) collected over a 1-year period (2006–2007) and full seasonal cycle in Mainz, Germany. In coarse particulate matter, we found 55 different hypothetical species (OTUs), of which 54 were plant pathogens and 29 belonged to the genus *Peronospora* (downy mildews). In fine particulate matter (<3 μm), only one species of *Hyaloperonospora* was found in one sample. Principal coordinate analysis of the species composition revealed three community clusters with a dependence on ambient temperature. The abundance of Oomycetes rRNA genes was low in winter and enhanced during spring, summer, and fall, with a dominance of *Phytophthora*, reaching a maximum concentration of ∼1.6 × 10^6^ rRNA genes per cubic meter of sampled air in summer. The presence and high concentration of rRNA genes in air suggests that atmospheric transport, which can lead to secondary infection, may be more important than currently estimated. This illustrates the need for more current and detailed datasets, as potential seasonal shifts due to changing meteorological conditions may influence the composition of airborne Oomycetes. An insight into the dynamics of airborne plant pathogens and their major drivers should be useful for improved forecasting and management of related plant diseases.

## Introduction

The dispersal of pathogenic microorganisms through the atmosphere has major implications for agriculture and public health. Some pathogens can travel over long distances, spreading diseases across and even between continents (Brown and Hovmoller, 2002; Burrows et al., 2009a,b; Womack et al., 2010; Després et al., 2012; Fisher et al., 2012; Fröhlich-Nowoisky et al., 2016).

Oomycetes are one of the most economically important and widespread group of plant pathogens. They are a diverse group of “fungus-like” eukaryotic organisms distributed globally in diverse environments and spreading through water, seeds, soil, and air (Göker et al., 2007; Dick, 2013; Beakes et al., 2015). Historically, Oomycetes were classified as fungi because of their similarities in hyphal organization and in nutrition (osmotrophy). However, since their molecular analysis in recent years, they have been redefined as stramenopiles (or heterokonts), to which brown algae also belong (Baldauf et al., 2000; Latijnhouwers et al., 2003; Andersen, 2004; Spring and Thines, 2004; Garcia-Blazquez et al., 2008; Riisberg et al., 2009; Dick, 2013; Beakes et al., 2015).

Oomycetes mainly spread as zoospores, wall-less free-swimming cells, which are released from sporangia during wet conditions (such as water splash, ground or underground water) and at temperatures below 12°C (Walker and van West, 2007; Dick, 2013). They can generate survival structures (formed for over-wintering, hot summer temperatures, or drought survival), i.e., thick-walled chlamydospores (asexual) and oospores (sexual) that are temperature-resistant (Dick, 1995; Fay and Fry, 1997; Vercesi et al., 1999; Fry and Grünwald, 2010; Crone et al., 2013). Oospores and sporangia can be dispersed through the air or attached to soil and plant particles (Kakde et al., 2001; Docampo et al., 2011; Mallo et al., 2011; Delmas et al., 2014; Manzano et al., 2015). Moreover, the spores are known to stay viable in soil up to 10 years (Judelson, 2008; Spencer-Phillips and Jeger, 2012). Under optimal growth conditions, these spores can germinate and infect host plants.

More than 60% of known Oomycetes species are plant pathogens, such as species from the families Albuginaceae (white blister rusts), Peronosporaceae (downy mildews), and Pythiaceae (Aylor et al., 1982; Göker et al., 2007; Walker and van West, 2007; Garcia-Blazquez et al., 2008; Voglmayr, 2008; Thines and Kamoun, 2010; Beakes et al., 2015). An understanding of their diversity, dynamics, and spreading behavior in the atmosphere on local and larger scales is important to improve infection risk prediction and disease management strategies (West et al., 2008). Improving monitoring and forecast of infection risk would also be advantageous for both economic and environmental reasons: curative treatment of already infected plants usually is more expensive and more stressful for the environment than treatments, which are applied before the disease actually infects the plants (Scholthof, 2006; Bebber and Gurr, 2015). However, for an improved disease forecasting and management, a more precise knowledge of the time of arrival, diversity, and abundance of infectious spores is necessary. Moreover, understanding the dynamics of plant pathogens and influences thereupon is important in food security and climate change (Pautasso et al., 2012).

Because of the parasitic nature of many Oomycetes species they are often not detected with standard culture-based methods (Arcate et al., 2006; Spring and Thines, 2010). Therefore, spore traps and microscopy combined with meteorological data are used to forecast sporulation and infection risk (West et al., 2008; Delmas et al., 2014). The development and application of DNA-based detection and quantification methods for airborne Oomycetes could provide a more accurate and unbiased monitoring, which could therefore lead to a better disease forecasting (Lévesque, 2011; Judelson, 2012). Furthermore, DNA-based methods enable the detection of target organisms or genetic changes in pathogen populations by choice of primers, which dependent on specificity allow the detection of all, some, or selected organisms in a sample (West et al., 2008).

Here, we combined DNA Sanger sequencing of the internal transcribed spacer (ITS) region with qPCR analysis of ribosomal RNA genes to investigate the species richness, rRNA gene abundance, and seasonal dynamics of airborne Oomycetes as well as their relationships with meteorological factors in continental air over a 1-year period.

## Materials and Methods

### Aerosol Sampling

Aerosol samples (57 pairs of fine and coarse particle samples) were collected on glass fiber filters (Pall Corporation, Dreieich, Germany, Type A/E, 102 diameter) over 1 year in Mainz, Germany (March 2006–April 2007) as described previously (Fröhlich-Nowoisky et al., 2009) and detailed in Supplementary Table 1.

Briefly, a self-built high-volume-dichotomous sampler (Solomon et al., 1983), was operated with a rotary vane pump (Becker, Wuppertal, Germany, Type VT 4.25) at a total flow rate of ∼0.3 m^3^min^-1^, corresponding to a nominal cut-off diameter of ∼3 μm. Thus, coarse particles with an aerodynamic diameter larger than ∼3 μm were collected on one glass fiber filter (∼0.03 m^3^min^-1^). The fine particles from the same air sample were collected on a second glass fiber filter (∼0.27 m^3^min^-1^). The sampling period was generally ∼7 days, corresponding to a sampled air volume of 3000 m^3^. A few samples were collected over shorter periods (volumes of ∼400–2000 m^3^). The sampling station was positioned on a mast about 5 m above the flat roof of the three-story high Max Planck Institute for Chemistry building located in the campus of the University of Mainz (49°59′31.36″N, 8°14′15.22″E). The sampled air masses represent a mix of urban and rural continental boundary layer air in central Europe. To ensure that the glass fiber filters were DNA free prior to sampling, they were baked overnight at 500°C. Loaded filters were packed in aluminum foil (baked at 500°C) and stored at -80°C until DNA extraction.

To detect possible contaminants from the sample handling or the sampler, blank samples were taken at 4-week intervals as previously described (Fröhlich-Nowoisky et al., 2009). Prebaked filters were mounted in the sampler, but the pump was either not activated (“mounting blanks”) or activated only for 5 s (“start-up blank”), respectively.

### DNA Extraction and Amplification

Filter aliquots (about ⅛−¼ of the filter) were extracted with a soil DNA extraction kit (LysingMatrixE, FastDNASpin Kit for Soil, MP Biomedicals, Eschwege, Germany) according to the supplier’s instructions with the following modifications. After lysis the mixtures were centrifuged for 10–15 min, partly followed by an addition of 900 μL buffer (kit-supplied) and a second repeat of bead-beating and centrifugation step. Both supernatants were combined for the further extraction process. Finally, the DNA was dissolved in 100 μL elution buffer. Extraction kit blanks containing no filter and baked filter blanks were included as extraction blanks.

For each DNA extract up to two PCR reactions were performed with the primer pair ITS4Oo/ITS5 and nested primer pairs ITS4Oo/ITS1 or ITS4/ITS5 (White et al., 1990; Nikolcheva and Bärlocher, 2004). The 50 μL reaction mixture contained 1–2 μL template DNA, 0.33 μM of each primer (Sigma-Aldrich, Munich, Germany), 1× JumpStart^TM^ PCR buffer (Sigma-Aldrich), 0.2 mM of each dNTP (Sigma-Aldrich) and 2.5 units of JumpStart^TM^ REDTaq DNA polymerase (Sigma-Aldrich). A negative control containing no template DNA was included in all PCR runs.

The thermal cycling conditions (DNA Engine, Bio-Rad Laboratories, Munich, Germany) consisted of an initial 3 min denaturation at 94°C, followed by 35 cycles of 30 s denaturation at 94°C, and 60 s annealing at 49°C (for ITS4Oo/ITS5 and ITS4Oo/ITS1) or 30 s at 54°C (ITS4/ITS5), proceeded with 90 or 45 s, respectively of elongation at 72°C and 3 min of final extension at 72°C.

PCR products were obtained for all coarse particle filter extracts and for six fine particle filter extracts. No DNA could be amplified from any of the six mounting, six start-up, 12 extractions, and 36 PCR-blanks, indicating that no contamination occurred during sample handling and analysis in the laboratory.

Amplification products for sequencing were cloned using the TOPO TA Cloning^®^ Kit (Thermo Fisher Scientific, Darmstadt, Germany) following the supplier’s instructions. Colonies containing inserts were identified by blue-white selection and lysed in 20 μL H_2_O for 10 min at 95°C. The inserts of 12–24 randomly picked colonies of each cloning reaction were amplified using 3 μL cell lysate in a 40 μL reaction. The PCR reaction mixture contained 1× PCR buffer (New England BioLabs, Frankfurt, Germany), 0.25 mM of each dNTP (New England BioLabs), 0.25 μM of each primer (Sigma-Aldrich) and 1.25 units of Taq DNA Polymerase (New England BioLabs). The PCR reactions were performed with the primer pair M13F-40 and M13R, and the thermal cycling conditions consisted of an initial 5 min denaturation at 94°C, followed by 40 cycles of 30 s denaturation at 94°C, 60 s annealing at 55°C, 60 s elongation at 72°C, and 15 min of final extension at 72°C. Up to 12 colony PCR products per original PCR product were sequenced.

The DNA sequences were determined with ABI Prism 377, 3100, and 3730 sequencers (Thermo Fisher Scientific) using BigDye-terminator v3.1 chemistry at the Max Planck-Genome-centre Cologne, Germany^1^. The quality of all sequences was manually checked and the vector sequences were cut. Out of 499 sequenced clones 30 sequencing reactions failed.

For comparison with known sequences, database queries using the Basic Local Alignment Search Tool (BLAST) were performed via National Center for Biotechnology Information (NCBI^2^). Each of the remaining 469 sequences was identified to the lowest taxonomic rank common to the top BLAST hits. Sixty sequences produced non-Oomycetes results and 6 sequences were assumed to be chimeric results of PCR recombination of the ITS1 and ITS2 regions and were excluded from further analysis. The Oomycetes DNA sequences were grouped into 55 OTUs (sequence identity scores ≥97%; Supplementary Table 2). Fifty-two OTUs, obtained by direct PCR amplification, were used for the species richness analysis, whereas three (OTU 31, 32, and 55, Supplementary Table 2) were excluded, as they were obtained by co-amplification of the bacterial 16S region (in an Acidiobacteria PCR with the primer pair Acid31/Eub518; Fierer et al., 2005). For each filter, sequences that produced the same BLAST results were pairwise aligned using the BioEdit program (BioEdit Sequence Alignment Editor 7.2.5^3^). Sequences with identity scores ≥97% were clustered into an operational taxonomic unit (OTU) and can be seen as hypothetical species as the intra-species variability is lower than 3% (Robideau et al., 2011), with a mean inter-species variability of 0.5% and a mean inter species variability of 30%. The sequences from the obtained OTUs of the present study have been deposited in the GenBank database with the accession numbers MF095126 – MF095180, detailed in Supplementary Table 3.

### Quantitative PCR

The rRNA genes of four selected Oomycete taxa and total Oomycetes were quantified from coarse particle filter extracts using the CFX96 quantitative PCR (qPCR) instrument (Bio-Rad Laboratories). Selection was based on taxa identified by Sanger-sequencing. The SYBR green method was used for *Pythium*, Albuginaceae, and *Peronospora* and for the total Oomycetes (Lang-Yona et al., 2012; Müller-Germann et al., 2015), and the TaqMan probe method for the *Phytophthora* (Kox et al., 2007) as specified in Table 1. All qPCR reactions were performed in triplicates of 10 μL mixtures. The SYBR green reactions contained 5 μL SsoAdvanced universal SYBR green supermix (Bio-Rad Laboratories), 1 μL extracted DNA, 500 nM of each primer (500 nM, Sigma-Aldrich), and 3 μL sterile, filtered water (Sigma-Aldrich). TaqMan probe reactions contained 5 μL SsoAdvanced universal probes supermix (Bio-Rad Laboratories), 1 μL extracted DNA, 500 nM of each primer (500 nM, Sigma-Aldrich) 200 nM probe (200 nM, Eurofins Genomics, Ebersberg, Germany), and 2.6 μL sterile, filtered water (Sigma-Aldrich).

**Table 1:** Primers used for qPCR.

Target	Amplified region	Primer name	Sequence	Amplicon length (bp)	Annealing temperature (°C)	Reference
Oomycetes	18S rRNA	AFP293-F	TTTCCGTAGGTGAACCTGCG	220–300	65	(Brouwer et al., 2003)
		AFP294-R	GCGAGCCTAGACATCCAC			
Albuginaceae	5.8S + 18S rRNA	Albug-F	GCTTCGGCTTGACACATTAG	93	62	(van Mölken et al., 2014)
		Albug-R	TCCGTCTCCTTGATGACCTT			
*Phytophtora*	18S rRNA	15Ph-F	TGCGGAAAGGATCATTACCACACC	248	60	(Kox et al., 2007)
		279Ph-R	GCGAGCCTAGACATCCACTG			
		All-Phy-P	FAM-TTGCTATCTAGTTAAAAGCA-TAMRA			
*Pythium*	18S rRNA	Pyth664-F	GCCCTTTCGGGTGTGTTACTAG	66	60	(Thomas et al., 2011)
		Pyth712-R	CTGAATGGCAGAAGAACATCCTC			
*Peronospora*	5.8S + 18S rRNA	Peron-F	CACGTGAACCGTATCAACC	98	62	(Hukkanen et al., 2006)
		Peron-R	GATAGGGCTTGCCCAGTAG			


*The amplified region, primer names, sequences, length,and annealing temperatures of the different qPCR primer sets for analysis of the total Oomycetes, Albuginaceae, Phytophthora, Pythium, and Peronospora.*

The thermal cycling conditions consisted of an initial 30 s or 2 min denaturation for SYBR green or TaqMan method, respectively, enzyme activation at 98°C, followed by 40 cycles of 10 s denaturation at 98°C, and 25 s annealing for both reaction types, and extension at primer pair specific temperatures as detailed in Table 1. The examination of the melt peaks confirmed amplification of the single desired product.

The qPCR results are reported in gene copy number (GCN) per cubic meter of air. Oomycete genes were not detected in mounting, start-up, extraction, and qPCR blanks, verifying that no contamination occurred during sample handling and analysis.

### gBlock DNA Fragments and qPCR Calibration

Calibration curves were derived using two self-designed gBlock DNA fragments (IDT, Iowa, United States). Fragment “gBlock A” contains two binding sites and “gBlock B” contains three binding sites for primer pairs detailed in Table 1 and illustrated in Supplementary Figure 1. Cross amplification of primer pairs was excluded using NCBI BLAST against Nucleotide collection (nr/nt) database. The designed amplicon size is based on the theoretical amplicon sizes of the selected taxa (Table 1).

Amplification efficiencies of the gBlock fragments calculated from standard curves of 10-fold dilutions (10^9^–10^1^ rRNA gene copies) were higher than 90% in all qPCR assays. Limits of quantification, as calculated from the standard curves were 2.5 ± 0.6, 1.8 ± 1.1, 1.4 ± 0.6, 1.3 ± 0.5, and 1.0 ± 0.1 gene copies for total Oomycetes, Albuginaceae, *Phytophthora, Peronospora*, and *Pythium*, respectively (Forootan et al., 2017).

### Meteorological Data

Local meteorological data (temperature, relative humidity (RH), atmospheric pressure, wind speed, and precipitation) were provided in half hour values by the ZIMEN Luftmessnetz, Rheinland-Pfalz, station Mainz-Mombach. The values were averaged for each sample period and are detailed in Supplementary Table 1.

### Statistical Analysis

The Pearson coefficient was calculated for correlations between the quantitative PCR values and meteorological factors, using OriginPro 9, to assess if there are potential significant linear correlations (*p*-value < 0.05).

Bray-Curtis (BC) dissimilarity in OTU composition was calculated for all aerosol filter pairs.

(1) $$BC_{ij}=1-\frac{2C_{ij}}{S_{i}+S_{j}}$$

Here *C_ij_* is the number of OTUs that two filter pairs *i* and *j* have in common, and *S* is the number of total OTUs (species richness) found on a filter pair *i* or *j*. A principle coordinate analysis (PCoA) was then performed on the resulting BC distances. Diversity clusters were identified using EM algorithm, using the R package mclust (vers.: 5.3) (Fraley and Raftery, 2002; Fraley et al., 2012).

## Results and Discussion

The species richness and rRNA gene abundance of airborne Oomycetes were investigated over a 1-year period and full seasonal cycle in Mainz, Germany, and correlated with meteorological factors to gain a better understanding of their seasonal dynamics in the atmosphere.

### Species Richness of Airborne Oomycetes

Oomycetes were identified by PCR amplicon Sanger sequencing on all 57 coarse particle filters, but only on one fine particle filter (Supplementary Table 2). This is consistent with previous observations of an enrichment of plant pathogenic fungi in the coarse particle fraction (Fröhlich-Nowoisky et al., 2009). A larger aerodynamic diameter is beneficial for a plant pathogen as a higher inertia will aid the impaction on a plants surface, thereby heightening the infection probability (Herries, 1961). Moreover, plant pathogens associated with aerosolized plant tissue fragments or soil particles will be enriched in the coarse particle fraction (Després et al., 2012, and reference therein).

The 55 identified OTUs comprise up to 3.6–6.1% of the estimated 900–1500 existing Oomycetes species (Arcate et al., 2006; Walker and van West, 2007; Dotzler et al., 2008; Voglmayr, 2008; Sandle, 2014). The Chao 2 estimator approach estimates an actual species richness of ∼79 species, indicating a coverage of approximately 70%. Moreover, rarefied and extrapolated estimates of the species richness estimate an even higher coverage, with a 95% confidence interval of 87.2–93.6% (Supplementary Figure 2). Of all identified OTUs, 54 were identified as pathogens (Supplementary Table 2). No information on the pathogenicity was found for the single OTU identified as *Pythium apiculatum.*

**FIGURE 1:**
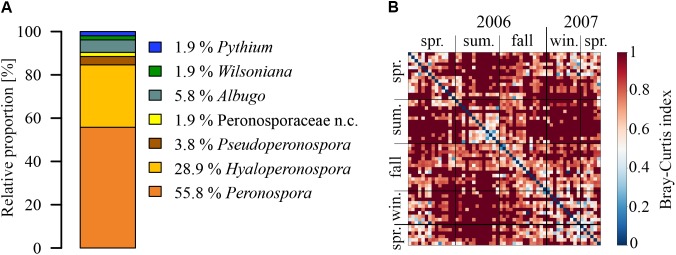
Species richness and seasonal dynamics in composition of airborne Oomycetes. **(A)** Relative proportions of different genera (n.c. = not classified), and **(B)** heatmap of Bray-Curtis (BC) dissimilarity in OTU composition between all analyzed samples. The BC index can vary between 0, for an identical OTU composition, and 1, for no common OTUs on the samples.

The detected OTUs were distributed over three families (Figure 1A). About 90% of the OTUs detected in coarse particle filters belonged to widespread obligate pathogenic family of Peronosporaceae to which all species of downy mildew belong. These pathogens are most commonly damaging grapes and a variety of vegetables (Gilles, 2004). The area surrounding the sampling site is predominately urban to the north and east, and dominated by agriculture with vineyards to the south and west. Small forests of ∼2–5 km^2^ can be found approximately 3.5–5 km in distance to the north-west and south west. Larger forests of over 100 km^2^ can be found to the north and east at a range of 10–15 km. From an agricultural perspective, the region is the largest wine growing area in Germany. The surrounding vineyards pose a potential major source for species of downy mildew and significant yield losses have been reported (Lieberz and Scott, 2017). Therefore, the Peronosporaceae detected on the air filters might stem from a local source. Moreover, the surrounding fractured landscape typical for northern Europe provides a multitude of natural and agricultural emission sources for the other Oomycetes genera with larger host spectra. The Peronosporaceae OTUs were assigned to three (out of 8 to 17) reported wind-dispersed genera (Riethmuller et al., 2002; Göker et al., 2004, 2007; Spencer-Phillips and Jeger, 2012), i.e., *Peronospora* (57%), *Hyaloperonospora* (29%), and *Pseudoperonospora* (4%). For one OTU, discernment between *Peronospora* and *Hyaloperonospora* was not possible. From the three families described above, 24% of the OTUs were identified down to the species level (Supplementary Table 2). The most abundant OTU, *Peronospora conglomerata*, a pathogen for geraniums (Farr and Rossman, 2017), was found on 30% of the coarse particle filters of all seasons. Albuginaceae (*Albugo* and *Wilsoniana*), to which many of white rusts belong, and Pythiaceae (*Pythium*), containing species known to cause root rot or damping off, represent 8% and 2% of the total identified OTUs, respectively.

Although the genus *Phytophthora* was expected in the air samples (Fall et al., 2015; Manzano et al., 2015), it was not detected by Sanger sequencing using the ITS primer pairs. However, it was the most abundant genus amongst the taxa quantified with qPCR. This is most probably because of a PCR selectivity of the general Oomycetes primers (albeit that the primers were previously reported to amplify *Phytophthora* spp.; Nikolcheva and Bärlocher, 2004). Further evidence for selectivity was a successful co-amplification of sequences that best matched *Phytophthora ramorum* (sequence similarity of 97%) using primers targeting the 16S rRNA of prokaryotes. This pathogen has a wide host range of 75 plant genera (e.g., oak, larch, and rhododendron). *Phytophthora ramorum* was found in samples that were mostly taken in periods of higher humidity and rain (apart from MZ 15) during fall (Supplementary Table 1). This corresponds to the spreading mechanism of *P. ramorum*, by airborne spores carried by wind-blown rain (Grunwald et al., 2008). The co-amplification in turn implies significant concentrations of airborne *P. ramorum* as it was the single oomycete identified amongst the abundant atmospheric bacterial population.

Seasonal dynamics of OTU composition of all analyzed samples is shown in Figure 1B. The BC dissimilarity index between two samples can vary between 1, indicating a completely different OTU composition, and 0, indicating an identical composition. Samples with half year differences, in general, show higher dissimilarities (values between 0.8 and 1) than samples in close temporal vicinity (along the diagonal) which show higher consistencies in OTU composition. This is not surprising in the seasonally changing climate of northern Europe as the plant pathogenic Oomycetes community structure will change with the annual vegetation cycle and will also change because of favorable meteorological conditions.

Samples taken at the late fall, winter, and spring show comparatively similar compositions. The summer samples, however, show a high sample-to-sample consistency (∼0.5 and lower), and a nearly completely different OTU composition than samples from other seasons, apart from a transitional phase in the first half of the fall season. This again can be explained by the vegetative cycle of host plants. Different species of Oomycetes are adapted to infect the different annual developmental stages of the hosts, such as leaves or fruits (Latijnhouwers et al., 2003; Thines and Kamoun, 2010). The high consistency between winter 2007 and the two spring seasons could be due to the aerosolization of soil particles during winter which contain soil dwelling Oomycetes species.

**FIGURE 2:**
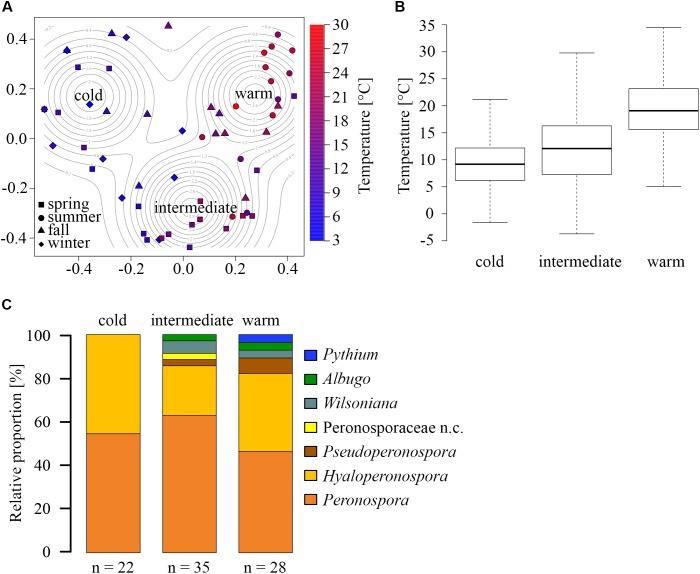
Temperature dependency of OTU composition. **(A)** Principle coordinate analysis (PCoA) of Bray-Curtis dissimilarities, revealing three temperature-dependent clusters with high, intermediate, and low average sampling temperatures. Point shape represents the sampling season, and color represents the average temperature for each aerosol filter pair. **(B)** Temperature distributions of the clusters (middle band: median, box: the 25 to 75th percentile, whiskers: 95% confidence interval. **(C)** Relative proportions of different genera within the three clusters (n.c. = not classified).

Principle coordinate analysis (PCoA) of the BC revealed three OTU community clusters (Figure 2A). These community clusters don’t seem to be primarily defined by a distinct seasonality (Figure 1B), but rather through the average ambient temperature during sampling (distinguished by point color) as each cluster contains samples from at least three seasons (distinguished by point shapes). Other meteorological parameter, such as mean relative humidity, wind speed, sum and duration of precipitation, did not show correlations with OTU clusters. Therefore, the clusters were named “warm”, “intermediate”, and “cold”. The mean temperatures for the different clusters are 9, 14, and 20°C (Figure 2B). Furthermore, the temperature distributions of the three clusters, analyzed by the Wilcoxon Rank test, were significantly different from another (*p*-value < 10^-16^). The cold cluster had the lowest number of OTUs (22), which all fell in the two genera *Hyaloperonospora* and *Peronospora* (Figure 2C). The intermediate and warm clusters display higher species richness with 35 and 28 OTUs, respectively. Taxonomically, both clusters are similar, with slight differences. The intermediate cluster contains the non-classified Peronosporaceae, and the warm cluster contains the only non-pathogenic OTU from the genus *Phytium*. A similar temperature dependency has been previously shown to influence the distribution and seasonal abundancies of various *Halophytophthora* species in river water (Nakagiri, 2000). The changing atmospheric community structure can be explained by two possible scenarios: A rapid response of the phyllospheric Oomycetes community composition, shifting the abundance toward other species, with changing temperatures, or an influence of temperature on the emission or sporulation process, of different species. Sporangia are very sensitive to mild changes in temperature (Byrt and Grant, 1979; Suzaki et al., 1996), and zoospore formation can be induced by a drop in ambient temperature (Hardham and Hyde, 1997).

### Ribosomal RNA Gene Abundance of Airborne Oomycetes

**FIGURE 3:**
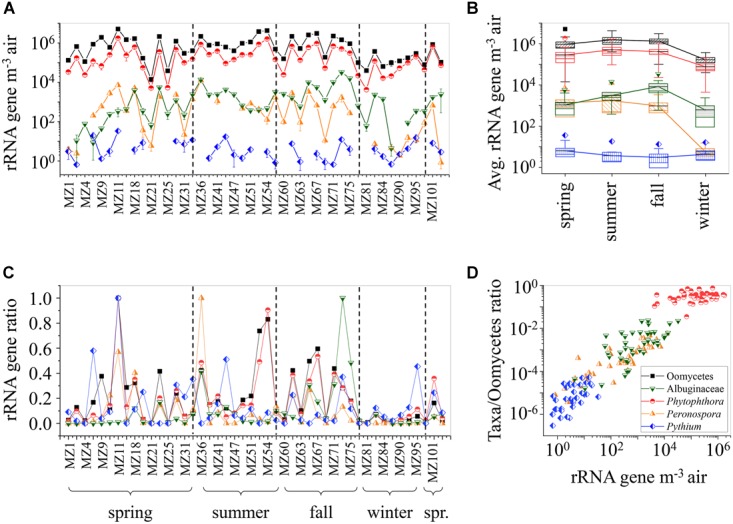
Oomycetes rRNA gene abundance retrieved from qPCR analysis. **(A)** The gene abundance (rRNA genes m^-3^ air) of selected taxa and total Oomycetes in coarse particle filter samples. Error bars represent standard deviation of triplicates. **(B)** Average seasonal rRNA gene abundance for selected taxa and total Oomycetes. Boxes limit 25 and 75% percentile, median presented as line, and mean values as point inside, connected in line. Error bars present 1 and 99% percentile. Outliers are shown (Student’s two sample *t*-test *p*-value < 0.01). **(C)** Gene abundance of selected taxa and total Oomycetes scaled to maximal taxon-specific values. **(D)** Gene abundance of selected taxa scaled to gene abundance of total Oomycetes, correlated with gene abundance of the selected taxa. Color codes in all panels: total Oomycetes marked as black squares, Albuginaceae as green inverted triangle, *Phytophthora* as red circles, *Peronospora* as orange triangles, and *Pythium* as blue diamond.

To evaluate the abundance and seasonal variation of airborne Oomycetes in coarse particulate matter, rRNA genes of selected taxa and total Oomycetes were quantified by qPCR with the primer pairs detailed in Table 1. The concentration of rRNA genes for total Oomycetes ranges between ∼1.4 × 10^4^ and ∼5.1 × 10^6^ GCN m^-3^ (Figure 3A and Supplementary Table 4). Highest concentrations of rRNA genes were observed for *Phytophthora* (ranging between 4.3 × 10^3^ and ∼1.8 × 10^6^ GCN m^-3^), whereas Albuginaceae and *Peronospora* had lower values (ranging between 0 and ∼3.3 × 10^4^, and 0 to ∼1.3 × 10^4^ GCN m^-3^, respectively). *Pythium* values were lowest (with maximum of ∼33 GCN m^-3^; MZ 11).

The seasonal averages of rRNA gene abundance are shown in Figure 3B. Total Oomycetes, *Phytophthora*, and *Peronospora* did not differ significantly during spring, summer, and fall, with highest levels observed during summer (∼1.6 × 10^6^ ± 7.0 × 10^5^, ∼4.9 × 10^5^ ± 2.4 × 10^5^, and ∼1.7 × 10^3^ ± 9.6 × 10^2^ GCN m^-3^, respectively). This corresponds to the seasons with the highest host plant availability in Europe, and conditions most beneficial for growth (Ellenberg, 2009). The Albuginaceae exhibited highest values in fall (∼8.1 × 10^3^ ± 5.0 × 10^3^ GCN m^-3^). No significant seasonal variation and low rRNA gene abundances were observed for *Pythium*. All other taxa had lowest concentrations in winter, with ∼1.7 × 10^5^ ± 6.2 × 10^4^, ∼6.0 × 10^2^± 4.5 × 10^2^, ∼7.3 × 10^4^± 3.5 × 10^4^, and ∼5.8 ± 2.9 GCN m^-3^ for total Oomycetes, Albuginaceae, *Phytophthora*, and *Peronospora*, respectively. Winter in central Europe is characterized by low vegetative yield (Ellenberg, 2009). Thus, a lower abundance of airborne plant pathogens can be expected. This finding is in concordance with previous studies of fungal spore abundance, reporting lower spore counts for lower outdoor temperatures (Tang, 2009, and references therein).

Disease warning systems for plant pathogenic Oomycetes are based on temperature and leaf wetness, with the assumption that certain leaf moistures and temperatures will allow efficient infection of plants (Abraham et al., 1995; Madden et al., 2000; Gilles, 2004; Henderson et al., 2007; Reis et al., 2013). Our results show a medium to high positive correlation of total Oomycetes rRNA GCN m^-3^ with RH during fall (See Supplementary Table 5). No significant correlations were observed with temperature and precipitation. The inconsistency between the correlations found in our study and the disease forecasting system might be due to different factors, such as the 7-day sampling periods used in this study. Furthermore, although plant infection risk correlates with RH and temperature (Palmieri et al., 2006; Li et al., 2014; Morales et al., 2018), atmospheric gene abundance of Oomycetes may not. The positive correlation with RH in fall could indicate preferential aerial transport under humid conditions, which is in agreement with previously reviewed dispersal of fungal and Oomycetes pathogens in tropical areas (Drenth and Guest, 2016).

As discussed in section “Species Richness of Airborne Oomycetes” the release and distribution of Oomycetes depends on ambient temperature, i.e., specific temperatures trigger release of certain species (Jones et al., 2014). Therefore, future studies should monitor and correlate Oomycetes abundance with temperature over shorter sampling times, or with extreme events. Climate change might lead to shift in spore release or species richness. Nevertheless, average annual air temperature in the investigated region is projected to increase by 1°C between 1990 and 2020 (Schröter et al., 2005). Other climatic factors such as precipitation are projected to have a mild change in this range of years. Therefore, we can assume that the species richness of airborne Oomycetes, detected in air samples collected in 2006, is still representative. The differential influence of meteorological factors on oospore release is outlined in Fry et al. (2008). Rain intensity and precipitation type (i.e., rain, drizzle, fog, etc.) could also affect the distribution of aerosolized Oomycetes. For example, prolonged rain will lead to wet deposition and therefore a washing of the atmosphere (Hemond and Fechner, 2015). However, short intensive rain may induce highly efficient release of oospores and Oomycetes fragments through a splash effect (Huffman et al., 2013), whereas a light drizzle could only have a minimal effect on aerosolization or deposition. Therefore, to estimate correlation with rain, further characterization of the precipitation type is required. Zoospores are free swimming in water films and can settle on surfaces and retract their flagella (Hardham and Hyde, 1997; Walker and van West, 2007). A subsequent secretion of a mucilaginous matrix affixes them to the surfaces, e.g., soil particles or leaf fragments which can then be aerosolized.

To further visualize common patterns in atmospheric presence, the rRNA gene abundance of the individual taxa was normalized by calculating the ratio to the highest taxon-specific value (Figure 3C). Common periodical tendencies with similar sample-to-sample dynamics are observed amongst all taxa. This again is most probably an indication of a passive influence affecting the atmospheric presence of all taxa, such as meteorological factors influencing emission and deposition. Additionally, in Figure 3D the gene abundance of the different taxa in individual air samples display a linear correlation with the gene abundance of total Oomycetes. The linear tendency indicates near constant proportions of single taxa within the total airborne Oomycetes, which might be due to dispersal mechanisms (e.g., attached to soil particles or plant fragments Sutton et al., 2006), rather than species-related emissions (e.g., wind-dispersed sporangia etc.).

Our results demonstrate the presence of plant-pathogenic Oomycetes over a 1-year period and full seasonal cycle in Mainz, Germany. Species composition analysis revealed occurrences of three plant-pathogenic families with seasonal dynamics and three community clusters with a dependence on ambient temperature. Higher concentrations of Oomycete rRNA genes in spring, summer, and fall, imply higher atmospheric transport rates in those seasons. The complementary input of the two methods combined in this study underlines the importance of parallel approaches in microbial ecology, where supportive analyses could help answering complex questions. Further investigations and monitoring of airborne Oomycetes, combining next generation DNA sequencing with quantitative approaches may be useful to gauge the current atmospheric Oomycetes community composition and correlations with meteorological conditions.

## Author Contributions

JF-N, NL-Y, and DP wrote the paper. JF-N, UP, VD, NL-Y, DP, DT, and JW designed the research. JF-N collected air samples and performed DNA extractions, cloning, and Sanger sequencing analysis. NL-Y and IM performed the qPCR analyses. DP, DT, and JW performed the statistical correlations and analysis with meteorological data. JF-N, UP, VD, NL-Y, DP, IM, DT, and JW discussed the results. All authors read and contributed to the manuscript.

## Conflict of Interest Statement

The authors declare that the research was conducted in the absence of any commercial or financial relationships that could be construed as a potential conflict of interest.

## Footnotes

1 http://mpgc.mpipz.mpg.de/home/

2 http://www.ncbi.nlm.nih.gov/

3 http://www.mbio.ncsu.edu/bioedit/bioedit.html
