# Corrigendum: Species Richness, rRNA Gene Abundance, and Seasonal Dynamics of Airborne Plant-Pathogenic Oomycetes

**DOI:** 10.3389/fmicb.2019.02200

**Published:** 2019-10-16

**Authors:** Naama Lang-Yona, Daniel A. Pickersgill, Isabel Maurus, David Teschner, Jörn Wehking, Eckhard Thines, Ulrich Pöschl, Viviane R. Després, Janine Fröhlich-Nowoisky

**Affiliations:** ^1^Multiphase Chemistry Department, Max Planck Institute for Chemistry, Mainz, Germany; ^2^Institute of Molecular Physiology, Johannes Gutenberg University Mainz, Mainz, Germany; ^3^Institute of Microbiology and Wine Research, Johannes Gutenberg University Mainz, Mainz, Germany

**Keywords:** airborne Oomycetes, Peronosporomycetes, plant pathogen, seasonal distribution, Sanger sequencing, qPCR analysis, meteorological parameter

There was an error in the Supplementary Material. The order of a few identified taxa did not match their OUT numbers. This does not require further changes in the main text file.

A correction has been made to Supplementary Table 2, Supplementary Table 3.

In addition, the phrase “similarity score” has been replaced with “sequence identity score” in the original article and the supplementary material.

**Supplementary Table 2:** Operational taxonomic units (OTUs) of identified Oomycetes. The OTU running number, frequency of occurrence (number of air samples in which the OTU was detected), best fitting NCBI accession numbers sorted by similarity score, taxonomic family, genus, and species name according to NCBI data base (if determined) and host plant families (numbers specify the corresponding host plant to the accession number of the pathogen). Species names are listed if available from NCBI sequences with sequence identity scores ≥ 97%.

**OTU**	**Frequency of occurrence**			**NCBI accession numbers and similarity scores**	**Family, genus, species**	**Host plant family of best match sequences**	**References**
	**Total**	**Coarse**	**Fine**				
1	20	20		AY198279 (100%), AY198276 (97%)	Peronosporaceae, *Peronospora* sp.	Caryophyllaceae	(Voglmayr, 2003)
2	17	17		AY198300 (100%), AY198301 (99%), AY198298 (98–99%)	Peronosporaceae, *Peronospora* sp.	Rubiaceae	(Voglmayr, 2003; Göker et al., 2007)
3	17	17		AY919304 (98–99%), AY198246 (99%)	Peronosporaceae, *Peronospora conglomerata*	Geraniaceae	(Voglmayr, 2003; Göker et al., 2007)
4	16	16		AY198244 (99%), AY198243 (97%)	Peronosporaceae, *Peronospora* sp.	Veronicaceae	(Voglmayr, 2003; Göker et al., 2007)
5	15	15		AY198243 (99%), AY198241 (99%)	Peronosporaceae, *Peronospora* sp.	Veronicaceae	(Voglmayr, 2003)
6	14	14		AF528557 (99–100%), AF528556 (99%), FM863723 (99%)	Peronosporaceae, *Peronospora* sp.	Amaranthaceae	(Byford, 1967; Choi et al., 2008)
7	15	14	1	JF975613 (99%), JF975614 (99%), AY210985 (99%)	Peronosporaceae, *Hyaloperonospora*	Brassicaceae	(Voglmayr, 2003; Göker et al., 2007)
8	11	11		EU049225.1 (100%), EU049249.1 (99%)	Peronosporaceae, *Hyaloperonospora* sp.	Brassicaceae	(Voglmayr, 2003; Göker et al., 2007)
9	9	9		EF174902 (99%), EF174889 (98–100%)	Peronosporaceae, *Peronospora* sp.	Fabaceae	(Voglmayr, 2003)
10	7	7		AY929824 (97–98%)	Albuginaceae, *Wilsoniana amaranthi*	Amaranthaceae	(Mirzaee et al., 2013)
11	6	6		AY198240 (99–100%)	Peronosporaceae, *Peronospora violae*	Violaceae	(Voglmayr, 2003)
12	6	6		KF888604 (99%), KF888591 (99%)	Peronosporaceae, *Peronospora* sp.	Amaranthaceae	(Klosterman et al., 2016)
13	5	5		EU295529 (99%), AY695806 (99%)	Peronosporaceae, *Peronospora arborescens*	Papaveraceae	(Voglmayr, 2003; Göker et al., 2007)
14	5	5		KC494997 (96%), KC494998 (96%)	Peronosporaceae, *Hyaloperonospora* sp.	Brassicaceae	(Voglmayr et al., 2014)
15	4	4		AY531462 (100%), EU049260 (100%)	Peronosporaceae, *Hyaloperonospora* sp.	Brassicaceae	(Voglmayr, 2003; Göker et al., 2007)
16	4	4		EU049263 (99%), AY578093 (99%)	Peronosporaceae, *Hyaloperonospora*	Brassicaceae	(Voglmayr, 2003; Göker et al., 2007; Coates and Beynon, 2010)
17	4	4		DQ447120 (99%), EU049273 (99%)	Peronosporaceae, *Hyaloperonospora* sp.	Brassicaceae	(Voglmayr, 2003; Göker et al., 2007)
18	3	3		EU660054 (99%), EF126356 (99–100%)	Peronosporaceae, *Pseudoperonospora* sp.	Balsaminaceae, Cannabaceae, Cucurbitaceae	(Choi et al., 2005; Voglmayr et al., 2009)
19	3	3		AY531452 (100%), AY210987 (99%)	Peronosporaceae, *Hyaloperonospora parasitica*	Brassicaceae	(Voglmayr, 2003; Göker et al., 2007)
20	3	3		EF174906 (99%), EF174949 (99%)	Peronosporaceae, *Peronospora* sp.	Fabaceae	(Voglmayr, 2003; Mouchacca, 2005)
21	3	3		KM058096 (96%), AY198248 (90%)	Peronosporaceae, *Peronospora* sp.	Veronicaceae	(Voglmayr, 2003; Göker et al., 2007)
22	2	2		AY531425 (100%), EU049276 (99%)	Peronosporaceae, *Hyaloperonospora* sp.	Brassicaceae	(Voglmayr et al., 2014)
23	2	2		GU583839 (100%), GU583838 (100%)	Peronosporaceae, *Hyaloperonospora erophilae*	Brassicaceae	(Voglmayr, 2003; Göker et al., 2007; Coates and Beynon, 2010)
24	2	2		EU049279 (99%), AY531452 (99%), AY198254 (99%)	Peronosporaceae, *Hyaloperonospora parasitica*	Brassicaceae	(Voglmayr, 2003; Göker et al., 2007)
25	2	2		KM058101 (95%), KM058095 (94%)	Peronosporaceae, *Peronospora* sp.	Ranunculaceae	(Riethmuller et al., 2002; Voglmayr, 2003; Voglmayr et al., 2014)
26	2	2		AY198280 (99%), KP271924 (98%)	Peronosporaceae, *Peronospora sp*.	1. Caryophyllaceae, 2. Polygonaceae	(Voglmayr, 2003; PetrŽelová et al., 2015)
27	2	2		EU049262 (99%)	Peronosporaceae, *Hyaloperonospora* sp.	Brassicaceae	(Voglmayr, 2003; Göker et al., 2007)
28	2	2		AY198307.1 (99–100%), HM636049.1 (99–100%), AY608613.1 (97–98%)	Peronosporaceae, *Pseudoperonospora* sp.	1-3. Urticaceae 3. Cannabaceae	(Voglmayr, 2003; Choi et al., 2005; Göker et al., 2007)
29	2	2		KC495031.1 (99%), EU049259.1 (99%)	Peronosporaceae, *Hyaloperonospora* sp.	Brassicaceae	(Voglmayr, 2003; Göker et al., 2007; Voglmayr et al., 2014)
30	2	2		KM058095 (97%), KM058101 (97%), KM058097 (97%)	Peronosporaceae, *Peronospora* sp.	Ranunculaceae	(Riethmuller et al., 2002; Voglmayr, 2003)
31	2	2		EU427470 (97%), DQ832718 (97%), DQ832717 (96%)	Peronosporaceae, *Phytophthora* sp.	1/2. Fagaceae, Ericaceae, Oleaceae, Theaceae, Adoxaceae; 3. Fabaceae	
32	2	2		EU427470 (96–97%), DQ832718 (96–97%), DQ832717 (96%)	Peronosporaceae, *Phytophthora* sp.		(Tyler et al., 2006; Grunwald et al., 2008)
33	1	1		KJ651417 (95%), HM636048 (94%), AY198307 (94%)	Peronosporaceae	Papaveraceae Urticaceae	(Voglmayr et al., 2014)
34	1	1		AY198293 (99%)	Peronosporaceae, *Peronospora valerianellae*	Valerianaceae	(Voglmayr, 2003)
35	1	1		HQ643443 (99%)	Pythiaceae, *Pythium apiculatum*		
36	1	1		AY211009 (99%), AY211010 (99%), EU049264 (99%)	Peronosporaceae, *Hyaloperonospora parasitica*	Brassicaceae	(Voglmayr, 2003; Göker et al., 2007)
37	1	1		AY210994.1 (98–99%), EU049210.1 (98–99%), AY198259.1 (98%)	Peronosporaceae, *Hyaloperonospora* sp.	Brassicaceae	(Voglmayr, 2003; Göker et al., 2007)
38	1	1		KM058095 (99%), KM058097 (99%), FJ384778 (98%)	Peronosporaceae, *Peronospora* sp.	Ranunculaceae	(Riethmuller et al., 2002)
39	1	1		AF241771 (100%)	Albuginaceae, *Albugo tragopogonis*	Asteraceae	(Long et al., 1975)
40	1	1		GQ390795 (99%), FJ394345 (99%)	Peronosporaceae, *Peronospora* sp.	Lamiaceae	(Thines et al., 2009; Henricot et al., 2010; Nagy and Horváth, 2011)
41	1	1		EU049207 (99%), EU049214 (99%)	Peronosporaceae, *Hyaloperonospora hesperidis*	Brassicaceae	(Göker et al., 2004; Voglmayr and Göker, 2011)
42	1	1		EF174893 (100%), EF174898 (99%)	Peronosporaceae, *Peronospora ervi*	Fabaceae	(Voglmayr, 2003)
43	1	1		AY198263 (99%), AY198264 (97%)	Peronosporaceae, *Peronospora* sp.	Boraginaceae	(Voglmayr, 2003; Göker et al., 2007)
44	1	1		KP271924 (97%), KM058096 (97%)	Peronosporaceae, *Peronospora* sp.	Polygonaceae	(PetrŽelová et al., 2015)
45	1	1		HM587262 (99%), FR825184 (99%)	Albuginaceae, *Albugo* sp.	Brassicaceae	(Kaur et al., 2011; Kemen et al., 2011)
46	1	1		KP271924 (95%), AY198279 (95%), AY198282 (94%)	Peronosporaceae, *Peronospora* sp.	1/3. Polygonaceae 2. Caryophylaceae	(Voglmayr, 2003; PetrŽelová et al., 2015)
47	1	1		AY198296 (99%), AY198295 (97%), AY198298 (95%)	Peronosporaceae, *Peronospora* sp.	1. Asteraceae 2. Lamiaceae 3. Rubiaceae	(Voglmayr, 2003)
48	1	1		DQ643921 (99%)	Albuginaceae, *Albugo portulacae*	Portulacaceae	(Choi et al., 2007)
49	1	1		HQ702191 88%), AY198247 (88%)	Peronosporaceae, *Peronospora* sp.	Lamiaceae, Scrophulariaceae	(Voglmayr, 2003; Nagy and Horváth, 2011)
50	1	1		AY198276 (97%), AY198277 (97%)	Peronosporaceae, *Peronospora* sp.	Caryophyllaceae	(Voglmayr, 2003)
51	1	1		AY198298 (94%), AY198296 (94%), AY198299 (94%)	Peronosporaceae, *Peronospora* sp.	1/3. Rubiaceae 2. Asteraceae	(Voglmayr, 2003)
52	1	1		AY198246 (96%), EU295529 (94%)	Peronosporaceae, *Peronospora* sp.	Geranicaceae, Papaveraceae	(Voglmayr, 2003; Göker et al., 2007)
53	1	1		AY198244 (95%), AY198243 (93%)	Peronosporaceae, *Peronospora* sp.	Veronicaceae	(Voglmayr, 2003; Göker et al., 2007)
54	1	1		AY198243 (95%), AY198241 (94%)	Peronosporaceae, *Peronospora* sp.	Veronicaceae	(Voglmayr, 2003; Göker et al., 2007)
55	1	1		EU427470 (97%), DQ832718 (97%), DQ832717 (96%)	Peronosporaceae, *Phytophthora* sp.	1/2. Fagaceae, Ericaceae, Oleaceae, Theaceae, Adoxaceae; 3. Fabaceae	(Tyler et al., 2006; Grunwald et al., 2008)

**Supplementary Table 3:** NCBI accession numbers of most representative sequences of the identified OTUs.

**OTU**	**NCBI accession number**	**OTU**	**NCBI accession number**
1	MF095133	29	MF095157
2	MF095126	30	MF095168
3	MF095129	31	MF095155
4	MF095132	32	MF095171
5	MF095135	33	MF095142
6	MF095127	34	MF095147
7	MF095134	35	MF095148
8	MF095156	36	MF095159
9	MF095128	37	MF095160
10	MF095131	38	MF095161
11	MF095136	39	MF095162
12	MF095139	40	MF095163
13	MF095140	41	MF095164
14	MF095145	42	MF095165
15	MF095130	43	MF095166
16	MF095146	44	MF095167
17	MF095158	45	MF095170
18	MF095137	46	MF095172
19	MF095138	47	MF095173
20	MF095149	48	MF095174
21	MF095153	49	MF095175
22	MF095141	50	MF095176
23	MF095143	51	MF095177
24	MF095144	52	MF095178
25	MF095150	53	MF095179
26	MF095151	54	MF095180
27	MF095152	55	MF095169
28	MF095154		

The authors apologize for this error and state that this does not change the scientific conclusions of the article in any way. The original article has been updated.

