# Supplementary material for: Species Richness, rRNA Gene Abundance, and Seasonal Dynamics of Airborne Plant-Pathogenic Oomycetes
Source: Front Microbiol. 2018 Nov 15;9:2673. doi: 10.3389/fmicb.2018.02673 (PMC6249755; doi:10.3389/fmicb.2018.02673)
Supplement: Supplementary file 1 [file Data_Sheet_1.pdf]

Supplementary Material

Species richness, gene abundance, and seasonal dynamics of plant-pathogen Oomycetes in continental air

Naama Lang-Yona[1,*,†], Daniel A. Pickersgill[1,2,†], Isabel Maurus[1], David Teschner[1,2], Jörn Wehking[1,2], Eckhard Thines[3], Ulrich Pöschl[1], Viviane R. Després[2], Janine Fröhlich-Nowoisky[1,*]

[1]Multiphase Chemistry Department, Max Planck Institute for Chemistry, Mainz, Germany
[2]Institute of Molecular Physiology, Johannes Gutenberg University, Mainz, Germany
[3]Institute of Microbiology and Wine Research, Johannes Gutenberg University, Mainz, Germany

*** Correspondence:**
Naama Lang-Yona and Janine Fröhlich-Nowoisky
n.lang-yona@mpic.de; j.frohlich@mpic.de

† These authors have contributed equally to this work.

1 Supplementary Figures and Tables

1.1 Supplementary Figures

A

TTAACGAGTTTTCCCAGTCACGATTTTTGGATCCATTGGAGGGCAAGTCTGGTGCCAGCAGCCGCGGTAATTCC

AFP293-F

AGCTCCAATAGCGTATATTTAAGTTT**TTTCCGTAGGTGAACCTGCG**GAAGGATCATTACCACACCTAAAAAA

ACTTTCCACGTGAACCGTTTCAACCAAATATTTTGGGGGTCTTGTCTGGCGTATGGCTGCTGCTGTAAAAGGCG

GCGGCTGTTGCTGGGTGAGCCCTATCATGGCGAACGTTTGGGCTTCGGTCTGAACAAGTAGCTCTTTTTTAAAC

CATTACTTATTACTGATTATACTGTGGGGACGAAAGTCTCTGCTTTTAACTAGATAGCAACTTTCAGCA**GTGGA**

AFP294-R *Pyth664-F*

TGTCTAGGCTCGCTTTTTGCCCTTTCGGGTGTGTTACTAGGATGTTTGAGACATTTTTTGTGAGGATGTTCTTC

Pyth712-R

TGCCATTCAGTTTTTGTCATAGCTGTTTCCTGGCGGCCGC

B

15Ph -F

TTAACGAGTTTTCCCAGTCACGATTTTTTGCGGAAAGGATCATTACCACACCTAATAATCTTTCCACTCAATCC

TACCAAGCCATTTCATTATGGGACTTGTACCACTATCATGGCGAATGTTTGACTTCGGTCCGGGCGAGTAGCTT

All-Phy -P

TATTGTTTTAAACCCATTTCACAATTCTGATTATACTGTGGGGACAAAAGTCTCTGCTTTTAACTAGATAGCAA

279Ph -R *Peron-F*

CTTTCAGCAGTGGATGTCTAGGCTCGCTTTTTCACGTGAACCGTATCAACCAACATATATTGGGGGTTTGTTTG

Peron-R *Albug-F*

TGCGGTGGCTGCTGTGCATTTTGTGCTGGCTGGCTGCTACTGGGCAAGCCCTATCTTTTGCTTCGGCTTGACAC

ATTAGCTCATGAATTGTATAGTTTGTTGTTCTTGTCAGAGTATACTTATGATAATCGAAAGGTCATCAAGGAGA

Albug-R

CGGATTTTTGTCATAGCTGTTTCCTGGCGGCCGC

Supplementary Figure 1. Self-designed gBlock fragments for qPCR calibration. The two fragments contain multiple primer binding sites for amplification of (A) total Oomycetes (shaded bold) and the genus *Pythium* (blue) and (B) for the genera *Phytophthora* (red) and *Peronospora* (orange) as well as for the family Albuginaceae (green).

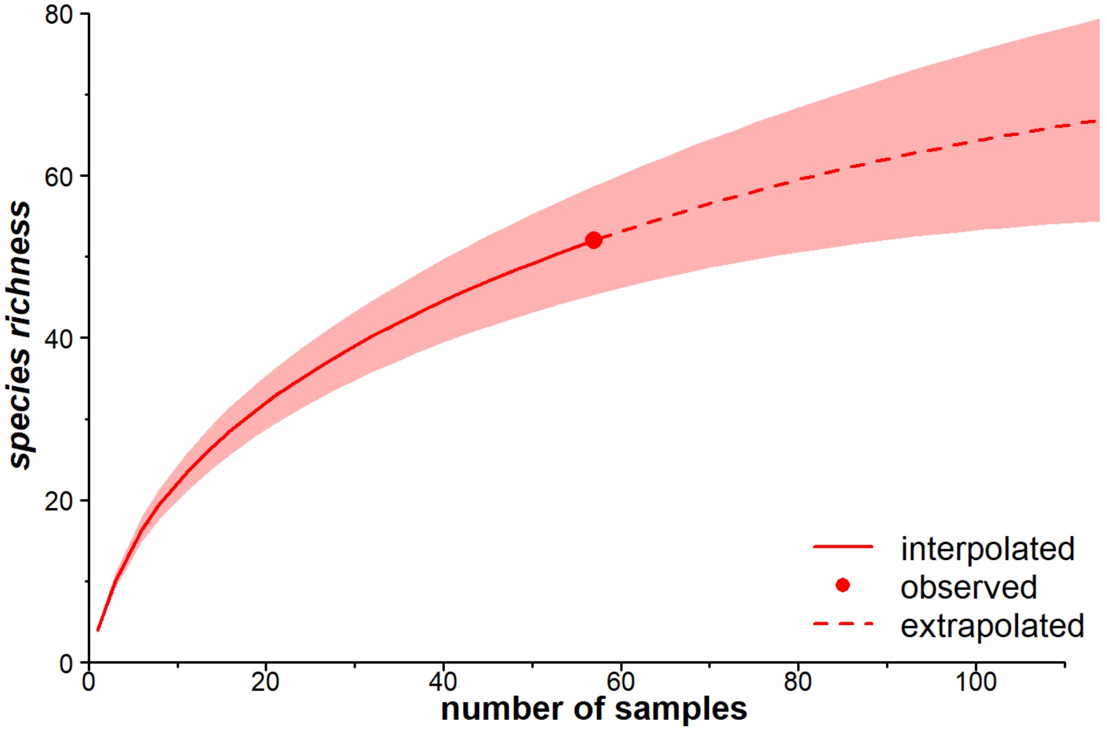

Supplementary Figure 2. Interpolated and extrapolated estimates of the Sanger sequencing OTU dataset, calculated and plotted with the R library iNext (Chao et al., 2014a;Chao et al., 2014b). The red shaded area corresponds to upper and lower 95% confidence interval.

1.2 Supplementary Tables

Supplementary Table 1. Overview of air samples (aerosol filter pairs) and meteorological parameters. Sample running number, sampling period (date, season), meteorological data (average temperature, relative humidity, atmospheric pressure, wind speed, precipitation), and sampled air volume for the aerosol filter pairs**.**

Sample number	Sampling period			Meteorological data					Sampled air volume
	Start	Stop	Season	Temp. Avg [°C]	RH Avg [%]	Atm. pressure Avg [hPa]	Wind speed Avg [m s^{-1}]	Precipitation Avg [L m^{-2}]	[m^3]
MZ 1	24.03.2006	31.03.2006	Spring	11.19	81.70	1009.87	2.00	26.74	3008.88
MZ 2	31.03.2006	07.04.2006	Spring	8.07	72.10	1015.84	1.97	14.76	3079.44
MZ 4	07.04.2006	12.04.2006	Spring	8.67	55.29	1015.30	2.02	5.29	2217.60
MZ 6	15.04.2006	18.04.2006	Spring	11.95	77.68	1012.93	2.17	6.37	1276.56
MZ 9	20.04.2006	27.04.2006	Spring	14.54	67.04	1017.90	1.39	11.54	3024.00
MZ 10	27.04.2006	02.05.2006	Spring	10.13	69.84	1016.26	1.98	6.33	2109.60
MZ 11	02.05.2006	03.05.2006	Spring	15.52	61.91	1017.49	1.03	0.00	438.12
MZ 15	04.05.2006	09.05.2006	Spring	19.25	45.58	1018.91	2.22	0.00	2102.40
MZ 18	12.05.2006	15.05.2006	Spring	16.81	69.36	1019.25	1.72	7.69	1311.12
MZ 19	15.05.2006	16.05.2006	Spring	19.51	63.30	1016.49	1.31	0.10	430.56
MZ 21	17.05.2006	18.05.2006	Spring	17.75	74.16	1016.44	1.22	1.65	429.12
MZ 24	18.05.2006	22.05.2006	Spring	14.41	73.83	1008.61	2.36	15.31	1742.40
MZ 25	22.05.2006	23.05.2006	Spring	13.21	80.40	1010.62	2.20	5.39	435.96
MZ 26	23.05.2006	30.05.2006	Spring	13.57	75.09	1019.08	2.23	27.93	3109.68
MZ 31	01.06.2006	06.06.2006	Spring	13.15	65.74	1025.79	1.87	3.01	2223.00
MZ 33	08.06.2006	13.06.2006	Spring	21.28	55.67	1025.75	1.45	0.00	2188.80
MZ 35	14.06.2006	21.06.2006	Spring	22.48	65.58	1017.24	2.43	1.82	3011.40
MZ 36	21.06.2006	22.06.2006	Summer	21.91	53.55	1014.24	1.65	0.00	433.44
MZ 40	27.06.2006	04.07.2006	Summer	23.29	59.85	1021.53	1.25	0.00	3061.80
MZ 41	04.07.2006	11.07.2006	Summer	23.97	67.91	1019.30	1.27	13.91	3036.60
MZ 42	11.07.2006	14.07.2006	Summer	29.20	63.21	1022.90	1.38	3.01	1312.20
MZ 43	14.07.2006	17.07.2006	Summer	23.79	45.99	1027.78	1.90	0.00	1324.08
MZ 45	19.07.2006	21.07.2006	Summer	28.64	53.47	1020.21	1.35	0.00	881.28
MZ 47	26.07.2006	02.08.2006	Summer	23.08	71.54	1013.62	1.52	23.00	3036.60
MZ 50	02.08.2006	09.08.2006	Summer	19.28	68.88	1014.38	1.61	22.88	3051.72
MZ 51	09.08.2006	16.08.2006	Summer	15.62	80.16	1010.92	1.21	18.85	3054.24
MZ 52	16.08.2006	23.08.2006	Summer	18.26	76.38	1014.82	1.13	33.84	3104.64
MZ 53	23.08.2006	30.08.2006	Summer	15.57	82.12	1011.54	1.42	34.91	3122.28
MZ 54	30.08.2006	06.09.2006	Summer	19.20	73.00	1021.07	1.08	2.69	1557.36
MZ 59	11.09.2006	18.09.2006	Summer	21.10	71.74	1012.82	1.12	2.36	3034.08
MZ 60	18.09.2006	25.09.2006	Fall	17.97	76.60	1014.94	1.72	0.03	3024.00
MZ 61	25.09.2006	02.10.2006	Fall	16.92	82.31	1014.43	1.73	22.64	3049.20
MZ 62	02.10.2006	09.10.2006	Fall	12.77	85.29	1015.46	1.44	38.7	3081.96
MZ 63	09.10.2006	16.10.2006	Fall	14.39	83.43	1024.34	1.07	2.53	3087.00
MZ 66	16.10.2006	23.10.2006	Fall	13.07	84.21	1009.55	1.80	1.78	3013.92
MZ 67	23.10.2006	30.10.2006	Fall	14.65	81.88	1015.59	1.16	12.91	3039.12
MZ 69	02.11.2006	09.11.2006	Fall	7.79	81.64	1028.56	2.11	3.30	3036.60
MZ 70	09.11.2006	16.11.2006	Fall	8.88	84.09	1019.66	2.50	4.81	3064.32
MZ 71	16.11.2006	23.11.2006	Fall	8.93	84.29	1011.15	3.05	9.87	3071.88

MZ 74	23.11.2006	30.11.2006	Fall	9.16	91.67	1018.99	1.24	3.15	2827.44
MZ 75	30.11.2006	07.12.2006	Fall	8.80	81.66	1016.26	1.24	2.75	3013.92
MZ 77	14.12.2006	21.12.2006	Fall	4.01	80.43	1031.53	2.22	3.64	3021.48
MZ 81	28.12.2006	04.01.2007	Winter	5.36	81.42	1024.74	1.36	16.65	2993.76
MZ 82	04.01.2007	11.01.2007	Winter	9.51	78.31	1017.84	2.45	4.54	3061.80
MZ 83	11.01.2007	18.01.2007	Winter	7.59	79.01	1023.32	1.50	7.88	3066.84
MZ 84	18.01.2007	25.01.2007	Winter	5.38	65.08	1013.88	1.43	13.16	3109.68
MZ 88	01.02.2007	08.02.2007	Winter	4.89	82.79	1020.55	2.00	12.25	3059.28
MZ 89	08.02.2007	15.02.2007	Winter	6.56	81.34	1005.79	1.97	29.66	3056.76
MZ 90	15.02.2007	22.02.2007	Winter	4.99	81.43	1018.14	2.02	0.11	2991.24
MZ 93	22.02.2007	01.03.2007	Winter	8.41	78.49	1007.47	2.17	22.93	3054.24
MZ 95	08.03.2007	15.03.2007	Winter	8.00	69.86	1031.51	1.39	3.62	3024.00
MZ 96	15.03.2007	22.03.2007	Winter	6.81	71.54	1011.54	1.98	9.84	3104.64
MZ 97	22.03.2007	29.03.2007	Spring	9.04	63.73	1015.17	1.03	14.16	3039.12
MZ 101	05.04.2007	12.04.2007	Spring	12.41	61.24	1022.94	2.22	0.00	3056.76
MZ 102	12.04.2007	19.04.2007	Spring	17.38	54.49	1021.59	1.72	0.00	3099.60
MZ 103	19.04.2007	26.04.2007	Spring	16.51	55.78	1020.48	1.31	0.00	3054.24

Supplementary Table 2. Operational taxonomic units (OTUs) of identified Oomycetes. The OTU running number, frequency of occurrence (number of air samples in which the OTU was detected), best fitting NCBI accession numbers sorted by similarity score, taxonomic family, genus, and species name according to NCBI data base (if determined) and host plant families (numbers specify the corresponding host plant to the accession number of the pathogen). Species names are listed if available from NCBI sequences with similarity scores ≥ 97%.

OTU	Frequency of occurrence			NCBI accession numbers and similarity scores	Family, genus, species	Host plant family of best match sequences	References
	total	coarse	fine				
1	20	20		AY198279 (100%), AY198276 (97%)	Peronosporaceae, *Peronospora* sp.	Caryophyllaceae	(Voglmayr, 2003)
2	17	17		AY198300 (100%), AY198301 (99%), AY198298 (98-99%)	Peronosporaceae, *Peronospora* sp.	Rubiaceae	(Voglmayr, 2003;Göker et al., 2007)
3	17	17		AY919304 (98-99%), AY198246 (99%)	Peronosporaceae, *Peronospora conglomerata*	Geraniaceae	(Voglmayr, 2003;Göker et al., 2007)
4	16	16		AY198244 (99%), AY198243 (97%)	Peronosporaceae, *Peronospora* sp.	Veronicaceae	(Voglmayr, 2003;Göker et al., 2007)
5	15	15		AY198243 (99%), AY198241 (99%)	Peronosporaceae, *Peronospora* sp.	Veronicaceae	(Voglmayr, 2003)
6	14	14		AF528557 (99-100%), AF528556 (99%), FM863723 (99%)	Peronosporaceae, *Peronospora* sp.	Amaranthaceae	(Byford, 1967;Choi et al., 2008)
7	15	14	1	JF975613 (99%), JF975614 (99%), AY210985 (99%)	Peronosporaceae, *Hyaloperonospora*	Brassicaceae	(Voglmayr, 2003;Göker et al., 2007)
8	11	11		EU049225.1 (100%), EU049249.1 (99%)	Peronosporaceae, *Hyaloperonospora* sp.	Brassicaceae	(Voglmayr, 2003;Göker et al., 2007)
9	9	9		EF174902 (99%), EF174889 (98-100%)	Peronosporaceae, *Peronospora* sp.	Fabaceae	(Voglmayr, 2003)
10	7	7		AY929824 (97-98%)	Albuginaceae, *Wilsoniana amaranthi*	Amaranthaceae	(Mirzaee et al., 2013)
11	6	6		AY198240 (99-100%)	Peronosporaceae, *Peronospora violae*	Violaceae	(Voglmayr, 2003)
12	6	6		KF888604 (99%), KF888591 (99%)	Peronosporaceae, *Peronospora* sp.	Amaranthaceae	(Klosterman et al., 2016)
13	5	5		EU295529 (99%), AY695806 (99%)	Peronosporaceae, *Peronospora arborescens*	Papaveraceae	(Voglmayr, 2003;Göker et al., 2007)
14	5	5		KC494997 (96%), KC494998 (96%)	Peronosporaceae, *Hyaloperonospora* sp.	Brassicaceae	(Voglmayr et al., 2014)
15	4	4		AY531462 (100%), EU049260 (100%)	Peronosporaceae, *Hyaloperonospora* sp.	Brassicaceae	(Voglmayr, 2003;Göker et al., 2007)
16	4	4		EU049263 (99%), AY578093 (99%)	Peronosporaceae, *Hyaloperonospora*	Brassicaceae	(Voglmayr, 2003;Göker et al., 2007;Coates and Beynon, 2010)
17	4	4		DQ447120 (99%), EU049273 (99%)	Peronosporaceae, *Hyaloperonospora* sp.	Brassicaceae	(Voglmayr, 2003;Göker et al., 2007)
18	3	3		EU660054 (99%), EF126356 (99-100%)	Peronosporaceae, *Pseudoperonospora* sp.	Balsaminaceae, Cannabaceae, Cucurbitaceae	(Choi et al., 2005;Voglmayr et al., 2009)
19	3	3		AY531452 (100%), AY210987 (99%)	Peronosporaceae, *Hyaloperonospora parasitica*	Brassicaceae	(Voglmayr, 2003;Göker et al., 2007)
20	3	3		EF174906 (99%), EF174949 (99%)	Peronosporaceae, *Peronospora* sp.	Fabaceae	(Voglmayr, 2003;Mouchacca, 2005)
21	3	3		KM058096 (96%), AY198248 (90%)	Peronosporaceae, *Peronospora* sp.	Veronicaceae	(Voglmayr, 2003;Göker et al., 2007)

22	2	2	AY531425 (100%), EU049276 (99%)	Peronosporaceae, *Hyaloperonospora* sp.	Brassicaceae	(Voglmayr et al., 2014)
23	2	2	GU583839 (100%), GU583838 (100%)	Peronosporaceae, *Hyaloperonospora erophilae*	Brassicaceae	(Voglmayr, 2003;Göker et al., 2007;Coates and Beynon, 2010)
24	2	2	EU049279 (99%), AY531452 (99%), AY198254 (99%)	Peronosporaceae, *Hyaloperonospora parasitica*	Brassicaceae	(Voglmayr, 2003;Göker et al., 2007)
25	2	2	KM058101 (95%), KM058095 (94%)	Peronosporaceae, *Peronospora* sp.	Ranunculaceae	(Riethmuller et al., 2002;Voglmayr, 2003;Voglmayr et al., 2014)
26	2	2	AY198280 (99%), KP271924 (98%)	Peronosporaceae, *Peronospora sp.*	1.Caryophyllaceae, 2.Polygonaceae	(Voglmayr, 2003;Petrželová et al., 2015)
27	2	2	EU049262 (99%)	Peronosporaceae, *Hyaloperonospora* sp.	Brassicaceae	(Voglmayr, 2003;Göker et al., 2007)
28	2	2	AY198307.1 (99-100%), HM636049.1 (99-100%), AY608613.1 (97-98%)	Peronosporaceae, *Pseudoperonospora* sp.	1-3.Urticaceae 3.Cannabaceae	(Voglmayr, 2003;Choi et al., 2005;Göker et al., 2007)
29	2	2	KC495031.1 (99%), EU049259.1 (99%)	Peronosporaceae, *Hyaloperonospora* sp.	Brassicaceae	(Voglmayr, 2003;Göker et al., 2007;Voglmayr et al., 2014)
30	2	2	KM058095 (97%), KM058101 (97%), KM058097 (97%)	Peronosporaceae, *Peronospora* sp.	Ranunculaceae	(Riethmuller et al., 2002;Voglmayr, 2003)
31	2	2	EU427470 (97%), DQ832718 (97%), DQ832717 (96%)	Peronosporaceae, *Phytophthora* sp.	Fagaceae, Ericaceae, Oleaceae, Theaceae, Adoxaceae; 3. Fabaceae	
32	2	2	EU427470 (96-97%), DQ832718 (96-97%), DQ832717 (96%)	Peronosporaceae, *Phytophthora* sp.		(Tyler et al., 2006;Grunwald et al., 2008)
33	1	1	KJ651417 (95%), HM636048 (94%), AY198307 (94%)	Peronosporaceae	Papaveraceae Urticaceae	(Voglmayr et al., 2014)
34	1	1	AY198293 (99%)	Peronosporaceae, *Peronospora valerianellae*	Valerianaceae	(Voglmayr, 2003)
35	1	1	HQ643443 (99%)	Pythiaceae, *Pythium apiculatum*		
36	1	1	AY211009 (99%), AY211010 (99%), EU049264 (99%)	Peronosporaceae, *Hyaloperonospora parasitica*	Brassicaceae	(Voglmayr, 2003;Göker et al., 2007)
37	1	1	AY210994.1 (98-99%), EU049210.1 (98-99%), AY198259.1 (98%)	Peronosporaceae, *Hyaloperonospora* sp.	Brassicaceae	(Voglmayr, 2003;Göker et al., 2007)
38	1	1	KM058095 (99%), KM058097 (99%), FJ384778 (98%)	Peronosporaceae, *Peronospora* sp.	Ranunculaceae	(Riethmuller et al., 2002)
39	1	1	AF241771 (100%)	Albuginaceae, *Albugo tragopogonis*	Asteraceae	(Long et al., 1975)
40	1	1	GQ390795 (99%), FJ394345 (99%)	Peronosporaceae, *Peronospora* sp.	Lamiaceae	(Thines et al., 2009;Henricot et al., 2010;Nagy and Horváth, 2011)
41	1	1	EU049207 (99%), EU049214 (99%)	Peronosporaceae, *Hyaloperonospora hesperidis*	Brassicaceae	(Göker et al., 2004;Voglmayr and Göker, 2011)
42	1	1	EF174893 (100%), EF174898 (99%)	Peronosporaceae, *Peronospora ervi*	Fabaceae	(Voglmayr, 2003)
43	1	1	AY198263 (99%), AY198264 (97%)	Peronosporaceae, *Peronospora* sp.	Boraginaceae	(Voglmayr, 2003;Göker et al., 2007)
44	1	1	KP271924 (97%), KM058096 (97%)	Peronosporaceae, *Peronospora* sp.	Polygonaceae	(Petrželová et al., 2015)
45	1	1	HM587262 (99%), FR825184 (99%)	Albuginaceae, *Albugo* sp.	Brassicaceae	(Kaur et al., 2011;Kemen et al., 2011)

46	1	1	KP271924 (95%), AY198279 (95%), AY198282 (94%)	Peronosporaceae, *Peronospora* sp.	1/3. Polygonaceae 2. Caryophylaceae	(Voglmayr, 2003;Petrželová et al., 2015)
47	1	1	AY198296 (99%), AY198295 (97%), AY198298 (95%)	Peronosporaceae, *Peronospora* sp.	1. Asteraceae 2. Lamiaceae 3. Rubiaceae	(Voglmayr, 2003)
48	1	1	DQ643921 (99%)	Albuginaceae, *Albugo portulacae*	Portulacaceae	(Choi et al., 2007)
49	1	1	HQ702191 88%), AY198247 (88%)	Peronosporaceae, *Peronospora* sp.	Lamiaceae, Scrophulariaceae	(Voglmayr, 2003;Nagy and Horváth, 2011)
50	1	1	AY198276 (97%), AY198277 (97%)	Peronosporaceae, *Peronospora* sp.	Caryophyllaceae	(Voglmayr, 2003)
51	1	1	AY198298 (94%), AY198296 (94%), AY198299 (94%)	Peronosporaceae, *Peronospora* sp.	1/3. Rubiaceae 2. Asteraceae	(Voglmayr, 2003)
52	1	1	AY198246 (96%), EU295529 (94%)	Peronosporaceae, *Peronospora* sp.	Geranicaceae, Papaveraceae	(Voglmayr, 2003;Göker et al., 2007)
53	1	1	AY198244 (95%), AY198243 (93%)	Peronosporaceae, *Peronospora* sp.	Veronicaceae	(Voglmayr, 2003;Göker et al., 2007)
54	1	1	AY198243 (95%), AY198241 (94%)	Peronosporaceae, *Peronospora* sp.	Veronicaceae	(Voglmayr, 2003;Göker et al., 2007)
55	1	1	EU427470 (97%), DQ832718 (97%), DQ832717 (96%)	Peronosporaceae, *Phytophthora* sp.	1/2. Fagaceae, Ericaceae, Oleaceae, Theaceae, Adoxaceae 3. Fabaceae	(Tyler et al., 2006;Grunwald et al., 2008)

Supplementary Table 3. NCBI accession numbers of most representative sequences of the identified OTUs.

OTU	NCBI accession number	OTU	NCBI accession number
1	MF095133	29	MF095157
2	MF095126	30	MF095168
3	MF095129	31	MF095155
4	MF095132	32	MF095171
5	MF095135	33	MF095142
6	MF095127	34	MF095147
7	MF095134	35	MF095148
8	MF095156	36	MF095159
9	MF095128	37	MF095160
10	MF095131	38	MF095161
11	MF095136	39	MF095162
12	MF095139	40	MF095163
13	MF095140	41	MF095164
14	MF095145	42	MF095165
15	MF095130	43	MF095166
16	MF095146	44	MF095167
17	MF095158	45	MF095170
18	MF095137	46	MF095172
19	MF095138	47	MF095173
20	MF095149	48	MF095174
21	MF095153	49	MF095175
22	MF095141	50	MF095176
23	MF095143	51	MF095177
24	MF095144	52	MF095178
25	MF095150	53	MF095179
26	MF095151	54	MF095180
27	MF095152	55	MF095169
28	MF095154		

Supplementary Table 4. Quantitative PCR results for rRNA genes (detailed in Table 1) of Oomycetes in coarse particulate matter. Filter sample running number, average rRNA gene abundance (gene copy number m^{-3}, n = 3 runs), and standard deviation (SD) for the total Oomycetes, Albuginaceae, *Phytophthora*, *Peronospora*, and *Pythium*.

Sample number	Season	Oomycetes Avg	Oomycetes SD	Albuginaceae Avg	Albuginaceae SD	*Phytophthora* Avg	*Phytophthora* SD	*Peronospora* Avg	*Peronospora* SD	*Pythium* Avg	*Pythium* SD
MZ 1	Spring	132647	8484	0.0	0.0	33623	3439	3.9	1.5	3.0	1.9
MZ 2	Spring	660248	21591	10.7	10.0	173014	2706	2.4	0.9	0.7	1.1
MZ 4	Spring	113338	15460	77.7	17.6	23865	5517	0.0	0.0	0.0	0.0
MZ 6	Spring	865499	50119	8.5	7.4	115448	47353	203.8	24.5	19.4	8.4
MZ 9	Spring	1938057	305849	44.7	8.1	67653	24690	596.1	48.0	1.3	2.3
MZ 10	Spring	606063	47769	237.3	61.9	252034	1393	2798.8	663.3	3.1	5.4
MZ 11	Spring	5144662	571473	336.7	162.5	1763896	94887	7147.6	1204.8	33.5	4.8
MZ 15	Spring	1476110	179703	427.2	42.8	232306	111720	353.2	25.2	0.0	0.0
MZ 18	Spring	1662387	140289	3917.0	732.2	619364	64675	5058.7	1107.4	3.7	6.5
MZ 19	Spring	168245	34162	349.2	126.4	55697	2645	36.3	20.2	8.4	14.5
MZ 21	Spring	14204	3734	65.7	23.8	5159	287	5.9	2.8	0.0	0.0
MZ 24	Spring	2139263	118944	5488.2	431.5	356575	55963	1824.6	121.1	0.0	0.0
MZ 25	Spring	38450	8292	248.8	120.1	5373	1272	0.0	0.0	0.0	0.0
MZ 26	Spring	1232300	68227	1198.3	154.7	461404	24784	2319.5	189.5	10.3	1.9
MZ 31	Spring	298467	19576	223.6	95.4	98818	5583	21.7	16.1	7.1	12.3
MZ 35	Spring	404940	12756	2450.4	86.3	151038	7119	1381.3	142.0	11.8	1.9
MZ 36	Summer	2177605	202571	13521.2	927.9	856443	51898	12541.0	2067.0	0.0	0.0
MZ 40	Summer	771889	65313	2333.8	346.7	260480	8349	0.0	0.0	1.4	2.4
MZ 41	Summer	898068	55655	2447.0	157.4	384782	32145	1095.7	328.7	5.2	0.6
MZ 45	Summer	624724	47700	4226.8	639.5	91053	5218	0.0	0.0	17.1	2.0
MZ 47	Summer	400480	39873	2437.5	223.6	144236	15927	0.0	0.0	2.1	3.6
MZ 50	Summer	963014	36186	657.3	54.8	251690	7361	466.2	78.0	1.4	2.5
MZ 51	Summer	1125035	61616	370.8	59.0	257359	20583	838.2	51.8	3.8	1.0
MZ 52	Summer	3795070	289972	399.0	84.8	866854	53189	421.4	68.0	0.0	0.0
MZ 54	Summer	4274819	386690	466.6	118.6	1595699	97904	125.0	49.0	2.9	1.4
MZ 59	Summer	488014	26877	3190.9	295.4	144514	18407	1687.7	220.8	0.8	1.4
MZ 60	Fall	157129	18839	2551.3	204.6	24311	4974	97.7	23.7	0.0	0.0
MZ 62	Fall	2173268	249307	1653.6	268.3	678461	34282	2785.7	391.4	7.6	1.6
MZ 63	Fall	526890	70651	580.9	109.2	134210	18534	90.3	56.7	0.9	1.6
MZ 66	Fall	2554584	317703	9871.4	766.9	596006	126244	3322.8	295.6	0.0	0.0
MZ 67	Fall	3063765	311458	5602.2	377.3	941664	8577	669.2	35.2	2.3	2.2
MZ 69	Fall	189725	5829	1246.2	177.8	52798	6570	11.0	0.0	0.9	1.5
MZ 71	Fall	2252326	343713	10124.8	148.2	693222	110423	615.7	150.2	0.7	1.2
MZ 74	Fall	1452678	112619	32519.3	1719.4	500398	35195	1659.2	661.9	12.4	2.8
MZ 75	Fall	920684	44942	15727.8	2814.3	306667	10006	269.4	22.4	3.9	2.0
MZ 77	Fall	100790	15436	637.0	137.6	21816	738	0.0	0.0	0.0	0.0
MZ 81	Winter	39722	1839	60.0	27.5	4270	1017	0.0	0.0	0.0	0.0
MZ 82	Winter	371992	36230	2298.2	174.5	119356	10380	7.3	1.8	4.1	1.8
MZ 84	Winter	64250	9658	1416.0	99.6	16360	1692	0.0	0.0	1.7	1.5
MZ 88	Winter	98477	32579	4.4	7.7	20999	249	4.6	0.5	0.7	1.2
MZ 90	Winter	121976	12513	0.0	0.0	53191	12016	1.9	3.3	2.2	0.5
MZ 93	Winter	176914	15957	87.2	6.0	98830	8827	15.1	5.6	4.3	0.3
MZ 95	Winter	292272	17911	349.7	33.2	199830	11312	11.6	3.5	15.2	6.4
MZ 97	Spring	79137	9529	337.7	34.9	43785	7617	88.0	33.8	0.0	0.0
MZ 101	Spring	837908	85860	1756.6	243.8	632423	60680	658.3	28.1	8.2	2.5
MZ 103	Spring	104578	15393	2453.5	2171.9	73155	2452	0.8	1.4	2.9	0.8

Supplementary Table 5. Correlation of Oomycetes rRNA gene abundance with temperature, relative humidity, and precipitation. Correlation coefficients for different taxa quantified by qPCR and seasons with temperature, relative humidity (RH), and precipitation. Correlations with winter samples are not presented due to the low number of qPCR-analyzed samples, low temperature range, lowest gene copy numbers, and insignificant correlations for this season.

	Temperature	RH	Precipitation
Albuginaceae			
Spring	0.332	-0.004	0.036
Summer	0.346	-0.723*	-0.500
Fall	-0.196	0.729*	-0.218
Phytophthora			
Spring	0.105	-0.148	-0.144
Summer	-0.361	0.139	-0.071
Fall	0.207	0.463	0.531
Peronospora			
Spring	0.169	-0.013	-0.046
Summer	0.005	-0.487	-0.331
Fall	0.195	0.534	0.472
Pythium			
Spring	0.209	0.003	-0.203
Summer	0.668*	-0.420	-0.308
Fall	0.195	0.534	0.472

*p-value < 0.05

2 References

Byford, W.J. (1967). Field experiments on sugar-beet downy mildew (Peronospora farinosa). *Annals of Applied Biology* 60**,** 97-107.

Chao, A., Chiu, C.H., Hsieh, T.C., Davis, T., Nipperess, D., and Faith, D. (2014a). *Rarefaction and extrapolation of phylogenetic diversity.*

Chao, A., Gotelli, N.J., Hsieh, T.C., Sander, E.L., Ma, K.H., Colwell, R.K., and Ellison, A.M. (2014b). Rarefaction and extrapolation with Hill numbers: a framework for sampling and estimation in species diversity studies. *Ecological Monographs* 84**,** 45-67.

Choi, Y.J., Denchev, C.M., and Shin, H.D. (2008). Morphological and molecular analyses support the existence of host-specific Peronospora species infecting Chenopodium. *Mycopathologia* 165**,** 155-164.

Choi, Y.J., Hong, S.B., and Shin, H.D. (2005). A re-consideration of Pseudoperonospora cubensis and P. humuli based on molecular and morphological data. *Mycol Res* 109**,** 841-848.

Choi, Y.J., Shin, H.D., Hong, S.B., and Thines, M. (2007). Morphological and molecular discrimination among Albugo Candida materials infecting Capsella bursa-pastoris world-wide. *Fungal Diversity* 27 I**,** 11-34.

Coates, M.E., and Beynon, J.L. (2010). Hyaloperonospora Arabidopsidis as a pathogen model. *Annu Rev Phytopathol* 48**,** 329-345.

Göker, M., Riethmüller, A., Voglmayr, H., Weiss, M., and Oberwinkler, F. (2004). Phylogeny of *Hyaloperonospora* based on nuclear ribosomal internal transcribed spacer sequences. *Mycological Progress* 3**,** 83-94.

Göker, M., Voglmayr, H., Riethmuller, A., and Oberwinkler, F. (2007). How do obligate parasites evolve? A multi-gene phylogenetic analysis of downy mildews. *Fungal Genet Biol* 44**,** 105-122.

Grunwald, N.J., Goss, E.M., and Press, C.M. (2008). *Phytophthora ramorum*: a pathogen with a remarkably wide host range causing sudden oak death on oaks and ramorum blight on woody ornamentals. *Mol Plant Pathol* 9**,** 729-740.

Henricot, B., Denton, J., Scrace, J., Barnes, A.V., and Lane, C.R. (2010). Peronospora belbahrii causing downy mildew disease on Agastache in the UK: a new host and location for the pathogen. *Plant Pathology* 59**,** 801-801.

Kaur, P., Sivasithamparam, K., and Barbetti, M.J. (2011). Host Range and Phylogenetic Relationships of Albugo candida from Cruciferous Hosts in Western Australia, with Special Reference to Brassica juncea. *Plant Disease* 95**,** 712-718.

Kemen, E., Gardiner, A., Schultz-Larsen, T., Kemen, A.C., Balmuth, A.L., Robert-Seilaniantz, A., Bailey, K., Holub, E., Studholme, D.J., Maclean, D., and Jones, J.D.G. (2011). Gene Gain and Loss during Evolution of Obligate Parasitism in the White Rust Pathogen of <italic>Arabidopsis thaliana</italic>. *PLoS Biol* 9**,** e1001094.

Klosterman, S., Mcroberts, N., and Subbarao, K.V. (2016). "Combined Annual Reports for Spinach and Lettuce Downy Mildew projects, 2015-2016".).

Long, D.E., Fung, A.K., Mcgee, E.E.M., Cooke, R.C., and Lewis, D.H. (1975). The Activity of Invertase and its Relevance to the Accumulation of Storage Polysaccharides in Leaves Infected by Biotrophic Fungi. *New Phytologist* 74**,** 173-182.

Mirzaee, M.R., Ploch, S., Runge, F., Telle, S., Nigrelli, L., and Thines, M. (2013). A new presumably widespread species of Albugo parasitic to Strigosella spp. (Brassicaceae). *Mycological Progress* 12**,** 45-52.

Mouchacca, J. (2005). Mycobiota of the arid Middle East: check-list of novel fungal taxa introduced from 1940 to 2000 and major recent biodiversity titles. *Journal of Arid Environments* 60**,** 359-387.

Nagy, G., and Horváth, A. (2011). Occurrence of Downy Mildew Caused by Peronospora belbahrii on Sweet Basil in Hungary. *Plant Disease* 95**,** 1034-1034.

Petrželová, I., Kitner, M., Jemelková, M., and Doležalová, I. (2015). First Report of Buckwheat Downy Mildew Caused by Peronospora cf. ducometi in the Czech Republic. *Plant Disease* 99**,** 1178.

Riethmuller, A., Voglmayr, H., Goker, M., Weiss, M., and Oberwinkler, F. (2002). Phylogenetic relationships of the downy mildews (Peronosporales) and related groups based on nuclear large subunit ribosomal DNA sequences. *Mycologia* 94**,** 834-849.

Thines, M., Telle, S., Ploch, S., and Runge, F. (2009). Identity of the downy mildew pathogens of basil, coleus, and sage with implications for quarantine measures. *Mycol Res* 113**,** 532-540.

Tyler, B.M., Tripathy, S., Zhang, X., Dehal, P., Jiang, R.H., Aerts, A., Arredondo, F.D., Baxter, L., Bensasson, D., Beynon, J.L., Chapman, J., Damasceno, C.M., Dorrance, A.E., Dou, D., Dickerman, A.W., Dubchak, I.L., Garbelotto, M., Gijzen, M., Gordon, S.G., Govers, F., Grunwald, N.J., Huang, W., Ivors, K.L., Jones, R.W., Kamoun, S., Krampis, K., Lamour, K.H., Lee, M.K., Mcdonald, W.H., Medina, M., Meijer, H.J., Nordberg, E.K., Maclean, D.J., Ospina-Giraldo, M.D., Morris, P.F., Phuntumart, V., Putnam, N.H., Rash, S., Rose, J.K., Sakihama, Y., Salamov, A.A., Savidor, A., Scheuring, C.F., Smith, B.M., Sobral, B.W., Terry, A., Torto-Alalibo, T.A., Win, J., Xu, Z., Zhang, H., Grigoriev, I.V., Rokhsar, D.S., and Boore, J.L. (2006). Phytophthora genome sequences uncover evolutionary origins and mechanisms of pathogenesis. *Science* 313**,** 1261-1266.

Voglmayr, H. (2003). Phylogenetic relationships of Peronospora and related genera based on nuclear ribosomal ITS sequences. *Mycol Res* 107**,** 1132-1142.

Voglmayr, H., Choi, Y.J., and Shin, H.D. (2014). Multigene phylogeny, taxonomy and reclassification of Hyaloperonospora on Cardamine. *Mycol Prog* 13**,** 131-144.

Voglmayr, H., and Göker, M. (2011). Morphology and phylogeny of Hyaloperonospora erophilae and H. praecox sp. nov., two downy mildew species co-occurring on Draba verna sensu lato. *Mycological Progress* 10**,** 283-292.

Voglmayr, H., Piątek, M., and Mossebo, D.C. (2009). Pseudoperonospora cubensis causing downy mildew disease on Impatiens irvingii in Cameroon: a new host for the pathogen. *Plant Pathology* 58**,** 394-394.
